# Associations between metabolic score for visceral fat and adult lung functions from NHANES 2007–2012

**DOI:** 10.3389/fnut.2024.1436652

**Published:** 2024-11-26

**Authors:** Jiacai Zhou, Linlin You, Xin Zhou, Yuying Li

**Affiliations:** ^1^Department of Respiratory and Critical Care Medicine, School of Clinical Medicine, Southwest Medical University, Luzhou, Sichuan, China; ^2^Inflammation & Allergic Diseases Research Unit, Department of Allergy, Affiliated Hospital of Southwest Medical University, Luzhou, Sichuan, China

**Keywords:** pulmonary function, cross-sectional study, NHANES, METS-VF, public health

## Abstract

**Background:**

Obesity is a significant part of the factors affecting lung function, and the assessment of obesity using the Metabolic Score for Visceral Fat (METS-VF) is more precise than other indicators like waist circumference and body mass index. This study investigated the relationship between lung function and METS-VF in The National Health and Nutrition Examination Survey (NHANES) database from 2007 to 2012.

**Method:**

The data utilized in this study was obtained from National Health and Nutrition Examination Survey spanning the years 2007 to 2012. A multivariate linear regression analysis was employed to investigate the association between METS-VF and lung function, followed by subgroup analysis to identify populations that may exhibit heightened sensitivity. Nonlinear correlations were assessed by fitting a restricted cubic spline, with validation of results conducted via threshold effect analysis.

**Result:**

In a study involving 4,356 participants, a weighted multiple linear regression model revealed a significant negative association between the METS-VF and forced expiratory volume in the first second (FEV1), forced vital capacity (FVC), FEV1/FVC ratio, and forced expiratory flow between 25 and 75% of FVC (FEF25-75%). However, no association was observed with peak expiratory flow rate (PEF). When dividing the METS-VF into thirds, participants in the highest third exhibited significantly decreased levels of FEV1 (*β*: −342, 95%CI: −440, −245, *p* < 0.001), FVC (*β*: −312, 95%CI: −431, −192, *p* < 0.001), FEV1/FVC (*β*: −0.020, 95%CI: −0.030, −0.010, *p* < 0.001), and FEF25-75% (*β*: −424, 95%CI: −562, −285, *p* < 0.001). However, there was no significant relationship with PEF (*β*: −89, 95%CI: −325, 147, *p* = 0.446). RCS curve indicated a nonlinear negative correlation between METS-VF and FEV1, FVC, and FEV1/FVC. For FEV1, a significant negative correlation was found when the METS-VF < 6.426 (*β* = −158.595, 95%CI: −228.183, −89.007). This negative association became more pronounced when the METS-VF > 6.426 (*β* = −314.548, 95%CI: −387.326, −241.770). For FVC, a negative association was observed when the METS-VF < 6.401, (*β* = −5.477, 95%CI: −91.655, 80.702), but it did not reach statistical significance. However, METS-VF > 6.401, METS VF and lung function show a significant negative correlation (*β* = −399.288, 95%CI: −486.187, −312.388). FEV1/FVC showed a negative correlation only before the inflection point (METS-VF < 6.263) (*β* = −0.040, 95%CI: −0.047, −0.032), after the inflection point (METS-VF > 6.263), no correlation was found, but there was no statistical significance (*β* = 0.000; 95%CI: −0.006, 0.007), and METS-VF had a linear negative correlation with FEF25-75%. Subgroup analysis showed that the association was consistent across a variety of demographic factors, including age, sex, race, hypertension, and coronary heart disease. In addition, we found a stronger association between men under 40 and lung function.

**Conclusion:**

METS-VF showed a linear negative correlation with FEF25-75%, and a nonlinear negative correlation with FEV1, FVC, FEV1/FVC, and FEF25-75%, but was not associated with PEF, particularly among males under the age of 40. These findings offer valuable insights into managing lung function by controlling visceral fat.

## 1 Introduction

Lung function, as a vital component of the human respiratory system, plays a pivotal role in facilitating gas exchange between the human body and the external environment, thereby ensuring the normal progression of essential life activities (1). The evaluation of pulmonary function is typically achieved through pulmonary function tests, such as measurable forced expiratory volume in 1 s (FEV1), forced vital capacity (FVC), FEV1/FVC ratio, forced expiratory flow between 25 and 75% of FVC (FEF 25–75%), and peak expiratory flow (PEF) (2). The leading causes of disability and mortality worldwide are respiratory disorders, such as chronic obstructive pulmonary disease (COPD) and asthma, which impose a significant societal and economic burden (3). Lung function has emerged as a pivotal diagnostic and evaluative tool for these chronic respiratory conditions (1). Currently, the escalating prevalence of obesity is deemed a significant public health concern (4). The prevailing consensus among various research indicates a robust correlation between obesity and compromised lung function, establishing it as a significant predisposing factor for respiratory ailments including chronic obstructive pulmonary disease, asthma, and pulmonary hypertension (5, 6), and an investigation demonstrated a robust association between increased indices related to obesity and a notable decline in pulmonary function over the course of the follow-up period (7). These studies collectively underscore the pivotal role of weight management and reduction in augmenting pulmonary function and respiratory health.

Body mass index (BMI) is commonly utilized for the classification of overall obesity and offers the advantage of assessing the severity of obesity. However, its specificity is limited as it fails to distinguish between lean body mass and adipose tissue mass, nor does it account for regional variations in fat distribution (8). In contrast, visceral obesity exerts a more pronounced impact on lung mechanics and metabolic inflammation compared to peripheral obesity (9, 10). The gold standard for clinical assessment of visceral obesity is magnetic resonance imaging (MRI); however, its application in clinical prevention is hindered by the high cost of detection and complex procedures involved (11, 12). The concept of Metabolic Score for Visceral Fat (METS-VF) has been proposed in order to evaluate visceral adipose tissue (13), and it has demonstrated significant value in assessing the risk of systemic diseases (14, 15). Furthermore, it exhibits a stronger evaluative effect compared to other established indicators of visceral fat (13). However, there is still a limited understanding of the relationship between METS-VF and lung function. With this knowledge gap in mind, our study aims to analyze data from the National Health and Nutrition Examination Survey (NHANES) in order to investigate the correlation between METS-VF and lung function through a cross-sectional analysis.

## 2 Materials and methods

### 2.1 Study population

**Figure 1:**
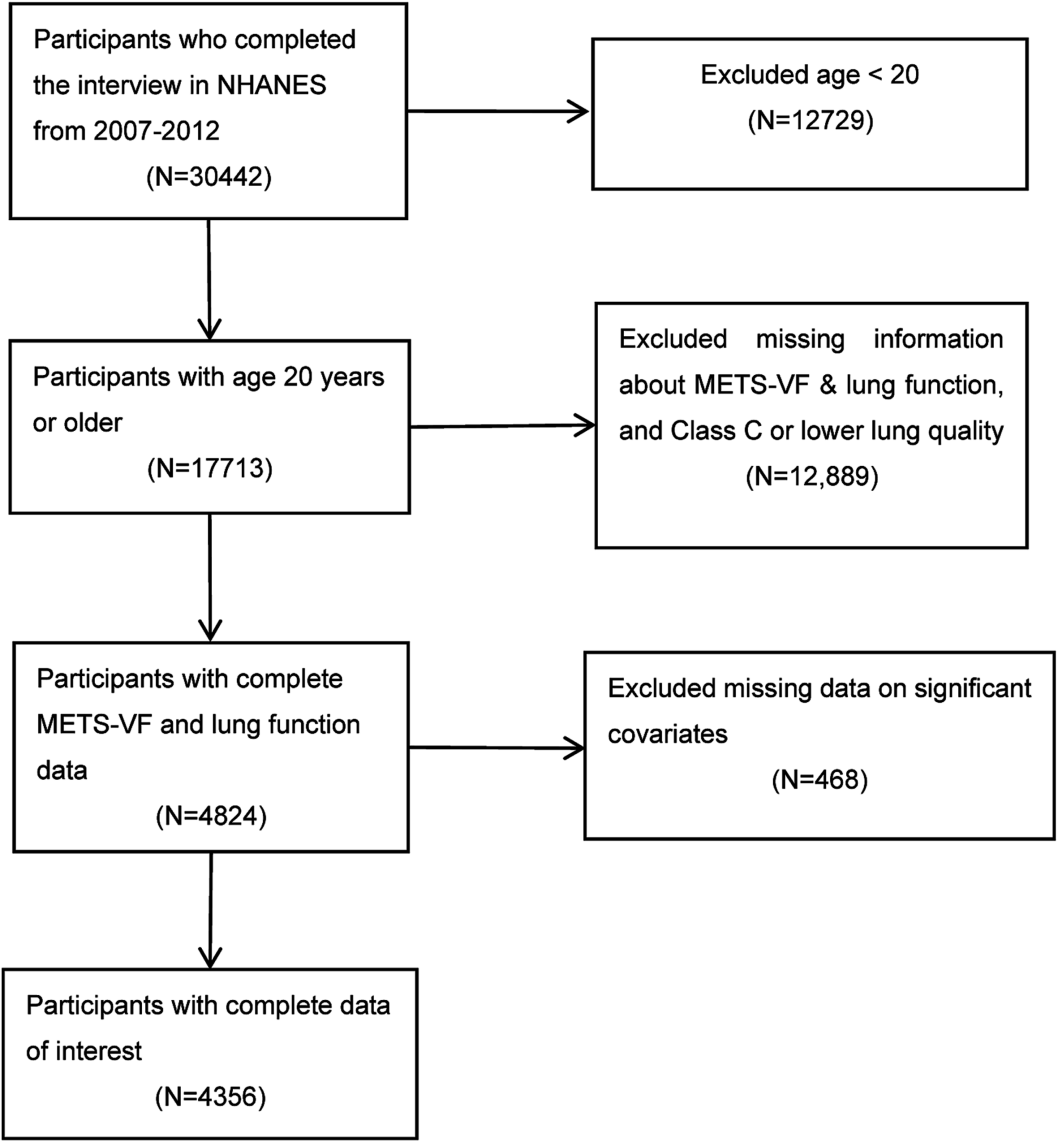
Flowchart of the sample selection from the 2007–2012 National Health and Nutrition Examination Survey (NHANES).

The NHANES database, overseen by the Centers for Disease Control and Prevention (CDC), aims to assess the health status of the US population and monitor trends in health risk factors. The participants were randomly selected using a sampling design that involved stratification, multiple stages, and probability-based selection. The data utilized in this cross-sectional study were obtained from NHANES surveys conducted during three cycles spanning 2007 to 2012, as these specific cycles included the collection of lung-function measurements. From an initial pool of 30,442 participants, we excluded individuals below the age of 20 (12,729 participants) as well as those with missing METS-VF data and lung function data rated C or lower on a categorical quality scale (12,889 participants). Additionally, we removed 468 individuals with incomplete information on significant covariates. Ultimately, our study included a total of 4,356 subjects (Figure 1). The NHANES initiative was approved by the research ethics review board at the National Center for Health Statistics (NCHS) and all participants provided written informed consent (16). Recruitment adhered strictly to the guidelines outlined in the Strengthening the Reporting of Observational Studies in Epidemiology (STROBE) statement (17). Relevant surveys and data are available on the NHANES website.

### 2.2 Measurement of metabolic score for visceral fat

The METS-VF, includes BMI, waist-to-height ratio (WHtR), fasting plasma glucose (FPG), high-density lipoprotein cholesterol (HDL-C), triglycerides (TG), age, and sex. The formula for METS-VF was as follows: METS-VF = 4.466 + 0.011*(Ln(METS-IR))^3^ + 3.239*(Ln(WHtr))^3^ + 0.319 * (Gender) + 0.594* (Ln(Age)), where METS-IR was calculated as Ln[(2 × fasting glucose+fasting triglycerides) × BMI]/[Ln(high-density lipoprotein cholesterol)]; Glucose was expressed in mg/dL, TG in mg/dL, BMI in kg/m^2^, HDL-C in mg/dL, age in years, and gender as a binary response variable (male =1, female =0).

### 2.3 Lung function measures

The age range of the subjects who underwent pulmonary function tests varied from 6 to 79 years. Key exclusion criteria encompassed recent instances of chest pain and difficulty breathing, the use of additional oxygen, ongoing surgeries involving the chest, abdomen, or eyes, as well as recent occurrences of stroke or heart attack. The testing procedures followed the recommendations established by the American Thoracic Society (ATS), while each healthcare technician received formal training. In this study, We used baseline spirometry data from the initial trial, and the pulmonary function measures that were necessary included FEV 1, FVC, FEF25-75%, and PEF. To ensure data accuracy, we exclusively utilized data with quality grades A and B.

### 2.4 Covariate definitions

Potential covariates included age, sex, race, BMI, education level, the poverty-to-income ratio (PIR) and physical activity, smoking status, alcohol consumption status, serum uric acid level, and disease status. PIR was divided into three levels: low income (PIR < 1), middle income (1 ≤ PIR ≤ 3), and high income (PIR > 3). Physical activity was categorized as vigorous (vigorous work and recreation), moderate (moderate work and recreation), or less than moderate (neither of these categories). The smoking status was classified into three categories: never smoked (lifetime <100 cigarettes), former smoker (lifetime >100 cigarettes, currently not smoking), and current smoker (> 100 cigarettes, occasionally or daily). History of alcohol use was confirmed by confirming whether participants consumed 12 or more alcoholic beverages in a year. Hypertension was characterized as having an average diastolic blood pressure of 80 mmHg or higher, or average systolic blood pressure of 130 mmHg or higher, or self-reported hypertension, or the use of medication to manage high blood pressure. Individuals were classified as having diabetes if their fasting blood glucose level exceeded 7 mmol/L, or if their random blood glucose level was above 11 mmol/L, or if their oral glucose tolerance test result after 2 h surpassed 11.1 mmol/L, or if their glycated hemoglobin level was higher than 6.5%, or if they self-reported having diabetes or used antidiabetic medications. In the health survey, individuals were inquired about whether a doctor or other healthcare professional has ever diagnosed them with coronary heart disease (CHD). If the response was affirmative, it was determined that the person had CHD.

### 2.5 Statistical analysis

Continuous variables are reported as mean ± standard deviation before weighting, and categorical variables are expressed as number of people before weighting (weighted percentage). The statistical technique of weighted analysis of variance (ANOVA) was employed to analyze continuous variables, while the Chi-square test was utilized for categorical variables. Furthermore, we developed three weighted multiple linear regression models to investigate the correlation between the METS-VF index and pulmonary function. In Model 1, the covariates remain unadjusted. Model 2 incorporates adjustments for race, education, and PIR. Model 3 includes adjustments for race, education, PIR, physical activity, smoking status, alcohol use, blood uric acid levels, hypertension, diabetes status, and coronary heart disease. The relationship between the METS-VF and lung function was examined using a restricted cubic spline analysis. In situations where a nonlinear association is detected, we employed the likelihood ratio test to identify the inflection point, which represents the point of greatest disparity in effects before and after reaching a specific METS-VF value. Age, sex, race, hypertension, and diabetes status were analyzed as subgroups. In addition, we have added interaction tests to check for potential interactions. All analyses were conducted in R4.3.3 and Empower software, with a two-tailed *p* < 0.050 indicating statistical significance.

## 3 Results

### 3.1 Cross-sectional characteristics of the participants

A total of 4,356 individuals were enrolled in the study. Table 1 presents the key characteristics of the study sample. Within this group, participants had an average age of 45 ± 15.39 years, with males accounting for 49.88% and females comprising 50.12%. The majority of individuals identified as non-Hispanic white, constituting approximately 72.19% of the cohort. Moreover, around 64.4% possessed a high school education or higher. Participants exhibited an average FEV 1 of 3252.52 ± 890.84 mL, an average FVC of 4182.41 ± 1086.75 mL, and an average FEV 1/FVC ratio of 0.78 ± 0.08. Furthermore, the mean values for FEF 25–75% and PEF were reported as 3014.05 ± 1,276.21 mL/s and 8414.85 ± 2157.08 mL/s. The range of METS-VF tertiles 1 to 3 was 3.692–6.283 (Q1), 6.284–6.858 (Q2), and 6.859–8.224 (Q3), respectively. Compared to subjects with a higher METS-VF index, individuals in the first tertile exhibited significant improvements in FEV 1, FVC, FEV 1/FVC, and FEF25-75% (*p* < 0.050) while PEF did not differ significantly among the three groups (*p* = 0.600).

**Table 1:** Weighted baseline characteristics of participants.

Characteristic	Overall, *N* = 4,356	METS-VF			
		Q1	Q2	Q3	*p*-value
Age (years)	45.00 (15.39)	33.60 (12.28)	42.64 (13.77)	51.88 (13.73)	<0.001
Sex (%)					<0.001
Male	2,194.00 (49.88%)	386.00 (38.66%)	447.00 (43.86%)	1,361.00 (58.50%)	
Female	2,162.00 (50.12%)	572.00 (61.34%)	571.00 (56.14%)	1,019.00 (41.50%)	
Race (%)					<0.001
Mexican American	679.00 (7.42%)	96.00 (5.27%)	151.00 (7.51%)	432.00 (8.46%)	
Other Hispanic	447.00 (4.84%)	76.00 (4.44%)	116.00 (5.60%)	255.00 (4.65%)	
Non-Hispanic White	2,096.00 (72.19%)	473.00 (70.71%)	498.00 (73.24%)	1,125.00 (72.42%)	
Non-Hispanic Black	815.00 (9.86%)	186.00 (10.33%)	181.00 (9.04%)	448.00 (10.03%)	
Other/multiracial	319.00 (5.69%)	127.00 (9.24%)	72.00 (4.62%)	120.00 (4.45%)	
Education (%)					<0.001
Less than high school	986.00 (14.82%)	145.00 (10.40%)	209.00 (14.79%)	632.00 (17.05%)	
High school	981.00 (20.78%)	183.00 (16.64%)	234.00 (21.29%)	564.00 (22.59%)	
More than high school	2,389.00 (64.40%)	630.00 (72.96%)	575.00 (63.91%)	1,184.00 (60.37%)	
PIR (%)					0.019
<1	842.00 (12.70%)	224.00 (16.11%)	201.00 (13.10%)	417.00 (10.80%)	
1–3	1,776.00 (33.71%)	345.00 (29.87%)	398.00 (32.53%)	1,033.00 (36.22%)	
>3	1,738.00 (53.59%)	389.00 (54.02%)	419.00 (54.37%)	930.00 (52.98%)	
Physical activity (%)					<0.001
Vigorous	1,692.00 (43.42%)	525.00 (58.78%)	414.00 (42.72%)	753.00 (36.07%)	
Moderate	1,438.00 (33.61%)	252.00 (26.10%)	343.00 (36.05%)	843.00 (36.15%)	
Below moderate	1,226.00 (22.97%)	181.00 (15.12%)	261.00 (21.23%)	784.00 (27.78%)	
BMI (kg/m^2^)	28.60 (6.26)	22.54 (2.54)	26.59 (3.10)	32.63 (5.81)	<0.001
Smoking status (%)					<0.001
Never smoker	2,359.00 (55.10%)	568.00 (59.28%)	585.00 (55.95%)	1,206.00 (52.58%)	
Former smoker	1,059.00 (24.98%)	138.00 (17.46%)	184.00 (20.13%)	737.00 (31.17%)	
Current smoker	938.00 (19.92%)	252.00 (23.26%)	249.00 (23.92%)	437.00 (16.25%)	
Alcohol intake (%)					0.003
Yes	3,351.00 (81.79%)	774.00 (83.86%)	797.00 (85.04%)	1,780.00 (79.12%)	
Not	1,005.00 (18.21%)	184.00 (16.14%)	221.00 (14.96%)	600.00 (20.88%)	
Uric acid(mg/dl)	5.51 (1.35)	4.81 (1.19)	5.22 (1.16)	6.01 (1.32)	<0.001
Hypertension (%)					<0.001
Yes	2,050.00 (43.19%)	162.00 (14.71%)	339.00 (30.84%)	1,549.00 (63.62%)	
Not	2,306.00 (56.81%)	796.00 (85.29%)	679.00 (69.16%)	831.00 (36.38%)	
Diabetes (%)					<0.001
Yes	760.00 (12.46%)	20.00 (1.65%)	74.00 (4.34%)	666.00 (21.93%)	
Not	3,596.00 (87.54%)	938.00 (98.35%)	944.00 (95.66%)	1,714.00 (78.07%)	
Coronary heart disease (%)					<0.001
Yes	120.00 (2.29%)	3.00 (0.12%)	16.00 (1.66%)	101.00 (3.69%)	
Not	4,236.00 (97.71%)	955.00 (99.88%)	1,002.00 (98.34%)	2,279.00 (96.31%)	
FEV1(ml)	3,252.52 (890.84)	3,504.78 (795.66)	3,321.54 (908.55)	3,091.73 (893.68)	<0.001
FVC (ml)	4,182.41 (1,086.75)	4,374.52 (962.24)	4,283.80 (1,110.39)	4,035.55 (1,112.96)	<0.001
FEV1/FVC	0.78 (0.08)	0.80 (0.08)	0.78 (0.08)	0.77 (0.08)	<0.001
FEF25-75%(ml/s)	3,014.05 (1,276.21)	3,403.09 (1,199.82)	3,052.71 (1,236.29)	2,799.98 (1,285.58)	<0.001
PEF (ml/s)	8,414.85 (2,157.08)	8,448.50 (1,911.72)	8,480.01 (2,231.47)	8,365.43 (2,233.59)	0.6

All values are presented as proportion (%) or mean (standard deviation).

PIR, Poverty to income ratio; FEV1, forced expiratory volume in the first second; FVC, forced vital capacity; FEF25-75%, forced expiratory flow between 25 and 75% of FVC; PEF, peak expiratory flow rate.

### 3.2 Association between the METS-VF and lung function

The results of a weighted generalized linear regression model, adjusted for various covariates, are presented in Table 2 to evaluate the association between METS-VF and lung function. The METS VF showed a significant negative correlation with FEV1, FVC, FEV1/FVC, and FEF25-75% in all models. These correlations remained consistent even after adjusting for all covariates: FEV1 (*β* = −234, 95%CI: −289, 179), FVC (*β* = −201, 95%CI: −264, −138), FEV1/FVC (*β* = −0.02, 95%CI: −0.02, −0.01), and FEF25-75% (*β* = −323, 95%CI: −409, −236). And following the categorization of METS-VF into triquartiles, a consistent negative correlation was observed across all models, with increasing significance as METS-VF levels increased (P for trend<0.001). Notably, irrespective of whether METS-VF was treated as a continuous or categorical variable, no significant association between PEF and METS-VF was detected in any adjustment model(*p* > 0.050).

**Table 2:** Weighted multivariate linear regression models of METS-VF with pulmonary function.

Characteristic	Model 1 ^1^*β* (95% ^2^CI)	*p*-value	Model 2 ^1^*β* (95% ^2^CI)	*p*-value	Model 3 ^1^*β* (95% ^2^CI)	*p*-value
FEV1 (ml)	−265 (−311, −218)	< 0.001	−266 (−312, −221)	< 0.001	−234 (−289, −179)	< 0.001
Tertile 1	Reference		Reference		Reference	
Tertile 2	−183 (−290, −76)	0.001	−192 (−298, −86)	< 0.001	−166 (−252, −80)	< 0.001
Tertile 3	−413 (−504, −322)	< 0.001	−411 (−498, −323)	< 0.001	−342 (−440, −245)	< 0.001
P for trend	<0.001		<0.001		<0.001	
FVC (ml)	−217 (−274, −159)	< 0.001	−224 (−278, −169)	< 0.001	−201 (−264, −138)	< 0.001
Tertile 1	Reference		Reference		Reference	
Tertile 2	−91 (−217, 35)	0.154	−109 (−232, 15)	0.083	−99 (−196, −1.7)	0.046
Tertile 3	−339 (−455, −223)	< 0.001	−344 (−456, −232)	< 0.001	−312 (−431, −192)	< 0.001
P for trend	<0.001		<0.001		<0.001	
FEV1/FVC	−0.02 (−0.03, −0.02)	< 0.001	−0.02 (−0.03, −0.02)	< 0.001	−0.02 (−0.02, −0.01)	< 0.001
Tertile 1	Reference		Reference		Reference	
Tertile 2	−0.03 (−0.04, −0.02)	< 0.001	−0.03 (−0.04, −0.02)	< 0.001	−0.02 (−0.03, −0.01)	< 0.001
Tertile 3	−0.04(−0.04, −0.03)	< 0.001	−0.03 (−0.04, −0.03)	< 0.001	−0.02 (−0.03, −0.01)	< 0.001
P for trend	<0.001		<0.001		<0.001	
FEF25-75% (ml/s)	−398 (−464, −333)	<0.001	−394 (−463, −325)	<0.001	−323 (−409, −236)	< 0.001
Tertile 1	Reference		Reference		Reference	
Tertile 2	−350 (−513, −188)	<0.001	−350 (−516, −185)	< 0.001	−283 (−441, −124)	0.001
Tertile 3	−603 (−720, −486)	<0.001	−593 (−712, −473)	< 0.001	−424 (−562, −285)	< 0.001
P for trend	<0.001		<0.001		<0.001	
PEF (ml/s)	−35 (−155, 85)	0.562	−31 (−155, 93)	0.618	−14 (−143, 116)	0.830
Tertile 1	Reference		Reference		Reference	
Tertile 2	32 (−227, 290)	0.808	30 (−234, 295)	0.817	46 (−164, 257)	0.657
Tertile 3	−83 (−313, 147)	0.471	−68 (−301, 166)	0.562	−89 (−325, 147)	0.446
P for trend	0.379		0.467		0.393	

^1β = Effect value.^

^2CI = Confidence Interval.^

Model 1 was adjusted for no covariates; Model 2 was adjusted for race, education and PIR; Model 3 was adjusted for covariates in Model 2+ physical activity, smoking status, alcohol intake, cholesterol, uric acid, hypertension, diabetes, coronary heart disease.

METS-VF: Metabolism score for visceral fat.

FEV1, forced expiratory volume in the first second; FVC, forced vital capacity; FEF25-75%, forced expiratory flow between 25 and 75% of FVC; PEF, peak expiratory flow rate.

**Figure 2:**
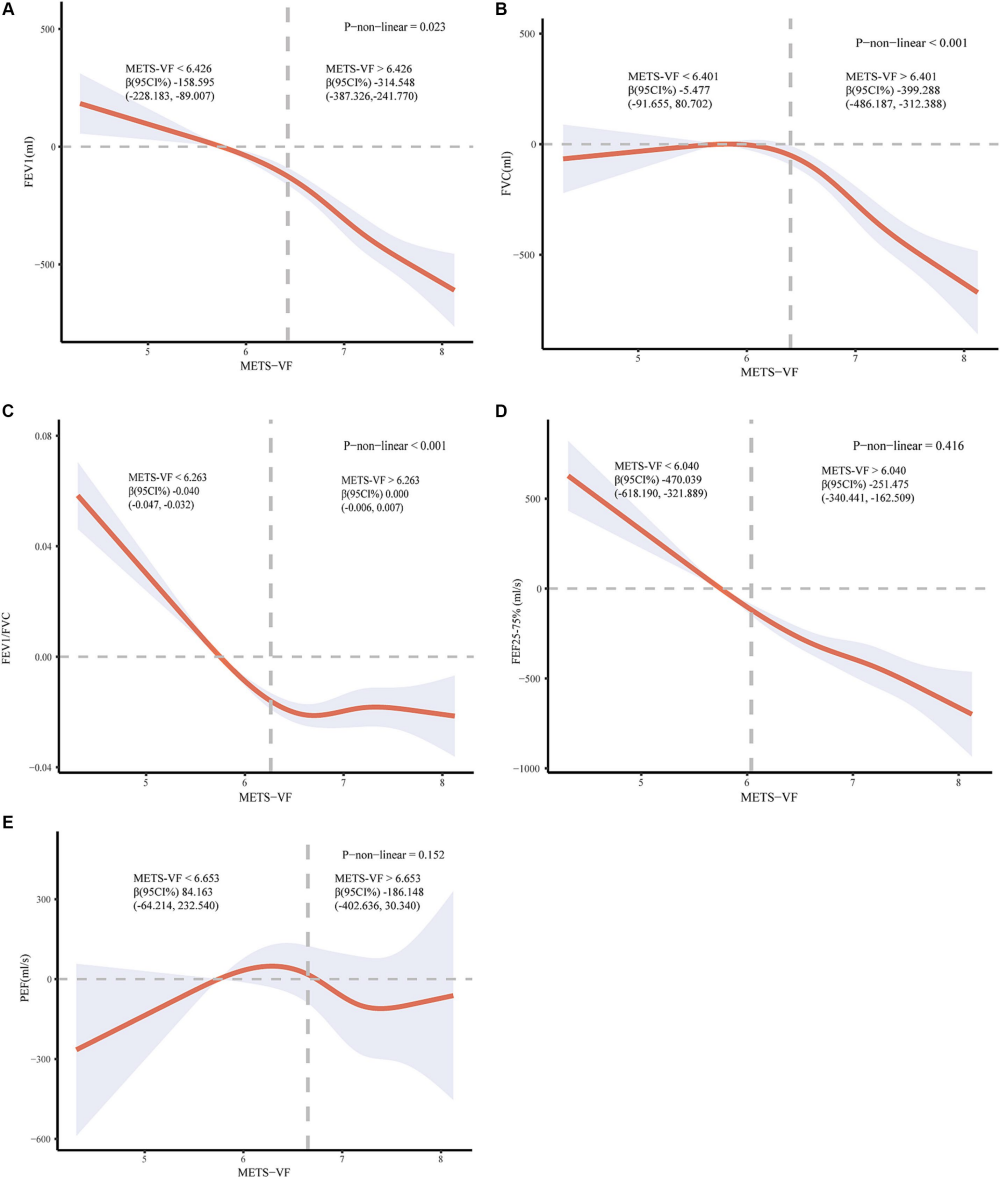
**(A)** Relationship between METS-VF and FEV1; **(B)** Relationship between METS-VF and FVC; **(C)** Relationship between METS-VF and FEV1/FVC; **(D)** Relationship between METS-VF and FEF25-75%. **(E)** Relationship between METS-VF and PEF. Adjusted for race, education, poverty-to-income ratio (PIR), physical activity, smoking status, alcohol use, blood uric acid levels, hypertension, diabetes status, and coronary heart disease.

The nonlinear relationship between METS-VF and lung function was analyzed by restricted cubic spline curve fitting (Figure 2). The multivariate-adjusted RCS curves demonstrated a significant non-linear inverse correlation trend (*p* < 0.050) between METS-VF and FEV1, FVC, as well as FEV1/FVC. Additionally, we conducted a threshold effect analysis (Supplementary material). For FEV1, a significant negative association was observed when the METS-VF was below 6.426 (*β* = −158.595, 95%CI: −228.183, −89.007). This negative association became more pronounced when the METS-VF index exceeded 6.426 (*β* = −314.548, 95%CI: −387.326, −241.770). Regarding FVC, we identified a negative association when the METS-VF index fell below 6.401 (*β* = −5.477; 95%CI: −91.655, 80.702), although statistical significance was not achieved in this case; however, for METS-VF indices above 6.401, there existed a significant and negative correlation between METS-VF and lung function (*β* = −399.288; 95%CI: −486.187, −312.388). Simultaneously, a negative correlation was observed between FEV1/FVC and METS-VF index only prior to the inflection point (METS-VF < 6.263) (*β* = −0.040; 95%CI: −0.047, −0.032). After surpassing the inflection point (METS-VF > 6.263), no significant correlation was found (*β* = 0.000; 95%CI: −0.006, 0.007). Regarding FEF25-75%, we identified a linear negative association with the METS-VF index (p-non-linear = 0.416), exhibiting consistent negative correlations before and after the inflection point; however, a more pronounced trend was observed before the inflection point (METS-VF < 6 0.04; *β* = −470.039; 95%CI: −618.190, −321.889) compared to after the inflection point (METS-VF >6 0.04; *β* = −251.475; 95%CI: −340.441, −162.509). Conversely, for PEF, a similar S-shaped pattern was observed in relation to the METS-VF according to RCS curve analysis, but statistical tests did not provide sufficient evidence supporting a significant linear or non-linear relationship (both *p* > 0.050).

### 3.3 Subgroup analysis

Subgroup analyses were conducted to further evaluate the stability of the association between METS-VF and lung function (Table 3). Interaction tests were performed, yielding the following findings: (1) the decline in lung function among male individuals becomes increasingly significant with an elevation in the METS-VF, particularly within those under 40 years old. Notably, race does not exert influence on the observed inverse correlation between FEV1, FVC, FEV1/FVC, FEF25-75%, and the METS-VF index. (2) The METS-VF index consistently correlated with FEV1, FEF25-75%, and PEF, and did not show significant associations with hypertension, diabetes, or coronary heart disease (p for interaction >0.050). (3) We found that the inverse association between METS-VF and FVC was not affected by coronary heart disease (p for interaction >0.050) and the effect size was more significant in the hypertensive population (*β* = −249, 95%CI: −342, −156). (4) The negative impact of the METS-VF on FEV1/FVC was found to be significant in patients without hypertension, diabetes, and coronary heart disease(*p* < 0.050); however, this effect exhibited an opposite trend in patients with diabetes. Nevertheless, it should be noted that the statistical significance of these findings was not particularly strong.

**Table 3:** Subgroup analysis for the association between METS-VF and pulmonary function.

Pulmonary function	Variables	*β* (95%CI)	*p*-value	P for interaction
FEV1 (ml)	Age(years)			<0.001
	<40	−132 (−196, −69)	<0.001	
	≥40	−6.6 (−94, 80)	0.9	
	Sex			<0.001
	Male	−360 (−429, −291)	<0.001	
	Female	−242 (−298, −186)	<0.001	
	Race			0.098
	Mexican American	−338 (−441, −234)	<0.001	
	Other Hispanic	−358 (−522, −194)	<0.001	
	Non-Hispanic White	−213 (−283, −144)	<0.001	
	Non-Hispanic Black	−316 (−418, −213)	<0.001	
	Other/multiracial	−199 (−352, −45)	0.016	
	Hypertension			0.233
	Yes	−242 (−298, −186)	<0.001	
	No	−273 (−336, −209)	<0.001	
	Diabetes			0.644
	Yes	−51 (−261, 159)	0.6	
	No	−267 (−320, −214)	<0.001	
	Coronary heart disease			0.147
	Yes	−149 (−597, 299)	0.5	
	No	−237 (−294, −180)	<0.001	
FVC(ml)
	Age(years)			<0.001
	<40	−96 (−165, −26)	0.009	
	≥40	−41 (−145, 63)	0.400	
	Sex			<0.001
	Male	−337 (−419, −254)	<0.001	
	Female	−239 (−296, −181)	<0.001	
	Race			0.023
	Mexican American	−341 (−464, −219)	<0.001	
	Other Hispanic	−398 (−579, −217)	<0.001	
	Non-Hispanic White	−228 (−306, −151)	<0.001	
	Non-Hispanic Black	−373 (−498, −249)	<0.001	
	Other/multiracial	−126 (−310, −58)	0.020	
	Hypertension			<0.001
	Yes	−249 (−342, −156)	<0.001	
	No	−222 (−299, −145)	<0.001	
	Diabetes			0.047
	Yes	−134 (−377, 109)	0.3	
	No	−234 (−293, −175)	<0.001	
	Coronary heart disease			0.212
	Yes	−84 (−766, 597)	0.8	
No	−206 (−271, −141)	<0.001		
FEV1/FVC	Age(years)			<0.001
	<40	−0.01 (−0.02, −0.01)	<0.001	
	≥40	0.01 (0.00, 0.02)	0.034	
	Sex			0.011
	Male	−0.02 (−0.03, −0.01)	<0.001	
	Female	−0.01 (−0.02, 0.00)	0.004	
	Race			0.870
	Mexican American	−0.02 (−0.04, −0.01)	<0.001	
	Other Hispanic	−0.02 (−0.03, −0.01)	0.004	
	Non-Hispanic White	−0.02 (−0.02, −0.01)	<0.001	
	Non-Hispanic Black	−0.01 (−0.02, 0.00)	0.120	
	Other/multiracial	−0.03 (−0.05, −0.01)	0.003	
	Hypertension			<0.001
	Yes	0.00 (−0.01, 0.01)	0.6	
	No	−0.02 (−0.03, −0.02)	<0.001	
	Diabetes			<0.001
	Yes	0.02 (0.00, 0.03)	0.037	
	No	−0.02 (−0.02, −0.01)	<0.001	
	Coronary heart disease			<0.001
	Yes	−0.01(−0.05, 0.03)	0.5	
	No	−0.02 (−0.02, −0.01)	<0.001	
FEF25-75%(ml/s)	Age(years)			<0.001
	<40	−154 (−257, −52)	0.004	
	≥40	85 (−24, 193)	0.12	
	Sex			<0.001
	Male	−474 (−590, −359)	<0.001	
	Female	−289 (−401, −178)	<0.001	
	Race			0.676
	Mexican American	−503 (−706, −300)	<0.001	
	Other Hispanic	−484 (−746, −221)	<0.001	
	Non-Hispanic White	−298 (−418, −178)	<0.001	
	Non-Hispanic Black	−343 (−511, −175)	<0.001	
	Other/multiracial	−390 (−645, −136)	0.006	
	Hypertension			0.149
	Yes	117 (−196, 430)	0.5	
	No	−370 (−461, −279)	<0.001	
	Diabetes			0.115
	Yes	26 (−293, 344)	0.9	
	No	−405 (−498, −312)	<0.001	
	Coronary heart disease			0.115
	Yes	−283 (−663,97)	0.13	
	No	−323 (−414, −232)	<0.001	
PEF(ml/s)	Age (years)			<0.003
	<40	118 (−46, 281)	0.200	
	≥40	311 (114, 508)	0.003	
	Sex			<0.001
	Male	−263 (−460, −66)	0.010	
	Female	−103 (−220, 14)	0.082	
	Race			0.004
	Mexican American	−180 (−519, 158)	0.300	
	Other Hispanic	−247 (−716, 223)	0.300	
	Non-Hispanic White	64 (−94, 222)	0.400	
	Non-Hispanic Black	−456 (−723, −190)	0.002	
	Other/multiracial	24 (−439, 486)	0.900	
	Hypertension			0.096
	Yes	−22 (−242, 199)	0.800	
	No	−77 (−227, 73)	0.300	
	Diabetes			0.096
	Yes	456 (−62, 974)	0.082	
	No	−97 (−220, 25)	0.220	
	Coronary heart disease			0.320
	Yes	99 (−1,309, 1,507)	0.900	
	No	−17 (−151, 118)	0.800	

## 4 Discussion

In this cross-sectional study, we investigated the association between the METS-VF and lung function in a population of U.S. adults aged 20 years or older using data from the NHANES collected between 2007 and 2012. We observed a robust negative correlation between the METS-VF and lung function parameters. The restricted cubic spline analysis revealed a non-linear negative relationship between METS-VF index and FEV1, FVC, and FEV1/FVC ratio, while a linear negative association was found with FEF25-75%. As the METS-VF increased, pulmonary function gradually declined; however, this effect varied across different measures of lung function. Specifically, an inverse trend with FVC was only evident beyond an inflection point, whereas no impact on FEV1/FVC was observed beyond a specific threshold. Furthermore, our findings indicated no significant correlation between METS-VF index and PEF. Subgroup analyses consistently demonstrated that these associations were independent of various demographic factors including age, sex, race as well as comorbidities such as hypertension and coronary heart disease. Notably, among men under 40 years old there existed a stronger link to impaired lung function.

Chronic respiratory diseases, such as asthma and chronic obstructive pulmonary disease (COPD), are closely associated with the global health burden. The examination of pulmonary function plays a crucial role in the early detection of lung and airway abnormalities, as well as in assessing disease severity and prognosis (1). Therefore, it is imperative to identify factors influencing lung function and implement early interventions for primary prevention of chronic respiratory diseases. Previous studies have indicated that abdominal obesity can serve as an indicator for early detection of changes in lung function (18, 19). Among individuals with abdominal obesity, visceral fat has been identified as the main determinant of impaired lung function (20). Although magnetic resonance imaging (MRI) technology and dual-energy X-ray absorptiometry (DXA) technology are currently available for accurate measurement of visceral fat in clinical practice, their high technical requirements and economic costs hinder large-scale implementation within public healthcare systems (8). BMI and WC have traditionally been used to assess abdominal obesity, however, these measures do not differentiate between visceral fat and subcutaneous adipose tissue (8). Bello-Chavolla OY et al. developed the METS-VF index which significantly outperforms these conventional indicators by demonstrating high consistency with gold standard measurements (13). Moreover, numerous research studies have indicated that the METS-VF demonstrates robust capability in evaluating and predicting the risk of metabolic disorders closely associated with visceral obesity, including chronic kidney disease, non-alcoholic fatty liver disease, and cardiovascular disease (21–23).

Previous studies have demonstrated a negative correlation between visceral fat and FVC as well as FEV1 (24). The study conducted by Qiushi Liu et al. involved 36,876 participants and observed a nonlinear positive correlation between the METS-VF index and asthma (25), which aligns with the clinical significance of our study — An increase in the METS-VF index may indicate a deteriorating lung function trend, ultimately leading to the development of chronic respiratory diseases. Our study reveals, for the first time, a negative correlation between an increase in the METS-VF index and FEV1, FVC, FEV1/FVC, and FEF25-75%, but no correlation with PEF. Additionally, we found that certain participants with specific characteristics were more likely to exhibit a negative association between METS-VF and lung function. Subgroup analysis demonstrated that this association was stronger in men than women, particularly among those under 40 years old; previous research also indicated that visceral fat was significantly associated with decreased lung function exclusively in men (26). Some studies have suggested that this could be attributed to higher BMI, larger waist circumference, and increased visceral fat in men (27), while women tend to experience an increase in total body and abdominal fat after menopause along with heightened insulin resistance (28). Hormonal differences may play a role as leptin regulates appetite and promotes fat burning while estrogen induces its production thereby limiting visceral adipose tissue accumulation (29), on the other hand, androgens not only inhibit leptin secretion but also enhance glucose uptake (30, 31).

Obesity causes significant changes in the mechanical properties of the lungs and chest wall due to fat accumulation in the mediastinum and abdominal cavity, resulting in decreased respiratory system compliance. This can lead to airway constriction, closure, increased resistance to breathing, and potential respiratory symptoms associated with obesity (32, 33). Adipose tissue immune cells are affected by obesity, leading to the production of pro-inflammatory substances (10). In a rodent model, mice fed a high-fat diet displayed an increase in body weight and accumulation of fat around the epididymis. They also showed impaired glucose tolerance and changes in their lipid profile. Furthermore, obese mice exhibited significantly higher levels of interleukin-5, eotaxin, tumor necrosis factor-*α*, and interleukin-10 in the fluid obtained from bronchoalveolar lavage compared to lean mice (34). In humans, these inflammatory mediators exhibit a strong association with systemic inflammation (35).

Our study possesses several strengths. Firstly, this is the first study to explore the association between METS-VF and lung function, conducting a comprehensive analysis of this relationship in a substantial sample size while considering five crucial indicators of lung function (FEV1, FVC, FEV1/FVC, FEF25-75%, and PEF). Moreover, our study utilizes an extensive and nationally representative cross-sectional dataset from the United States. We have implemented a weighting mechanism to fully account for sampling weight and control confounding factors, ensuring the precision and representativeness of our findings. Nevertheless, there are also certain limitations that necessitate consideration. Firstly, due to its cross-sectional design, establishing the causality or directionality of associations is not feasible. Secondly, this study only focused on the lung function of adults over 20 years old in the United States. In the future, it is necessary to discuss and analyze the data of other countries or regions to reduce the differences caused by population. Thirdly, as the METS-VF index is an innovative assessment measure, it remains uncertain whether obesity or wasting corresponds to its turning point. Lastly, specific variables such as physical activity level, hypertension diabetes, and coronary heart disease rely on self-reported data which may introduce recall bias.

## 5 Conclusion

In conclusion, our study demonstrated a robust association between METS-VF and the risk of decline in FEV1, FVC, FEV1/FVC, and FEF25-75%, while no significant association was observed with PEF after adjusting for potential confounders. Notably, for males under 40 years old, early implementation of weight control and management is recommended to enhance lung function and prevent the onset of chronic lung diseases. These findings warrant further validation through prospective studies in the future.

## Data Availability

Publicly available datasets were analyzed in this study. This data can be found at: https://www.cdc.gov/nchs/nhanes/index.htm.
