# Supplementary material for: Associations between metabolic score for visceral fat and adult lung functions from NHANES 2007–2012
Source: Front Nutr. 2024 Nov 26;11:1436652. doi: 10.3389/fnut.2024.1436652 (PMC11628286; doi:10.3389/fnut.2024.1436652)
Supplement: Supplementary file 1 [file Table_1.docx]

Supplementary material: Threshold effect analysis of MTES-VF

| Outcome | FEV1 | FVC | FEV1/FVC | FEF25-75% | PEF |
| --- | --- | --- | --- | --- | --- |
| Model I (linear fitting) | β(95CI%) -234.029  (-273.852,-194.205)  P<0.0001 | β(95CI%) -201.191  (-249.653,-152.728)  P<0.0001 | β(95CI%) -0.017  (-0.021, -0.013)  P<0.0001 | β(95CI%) -322.534  (-383.173, -261.895)  P<0.0001 | β(95CI%) -13.757  (-114.673, 87.158)  P = 0.7893 |
| Inflection point (K) | 6.426 | 6.401 | 6.263 | 6.040 | 6.653 |
| METS-VF < K | β(95CI%) -158.595  (-228.183, -89.007)  P<0.0001 | β(95CI%) -5.477  (-91.655, 80.702)  P = 0.9009 | β(95CI%) -0.040  (-0.047, -0.032)  P<0.0001 | β(95CI%) -470.039  (-618.190, -321.889)  P<0.0001 | β(95CI%) 84.163  (-64.214, 232.540)  P = 0.2663 |
| METS-VF > K | β(95CI%) -314.548  (-387.326,-241.770)  P<0.0001 | β(95CI%) -399.288  (-486.187, -312.388)  P<0.0001 | β(95CI%) 0.000  (-0.006, 0.007)  P = 0.9272 | β(95CI%) -251.475  (-340.441, -162.509)  P<0.0001 | β(95CI%) -186.148  (-402.636, 30.340)  P = 0.0920 |
| likelihood ratio test | 0.009 | <0.001 | <0.001 | 0.032 | 0.077 |
